# Evidence of MRSE on a gentamicin and vancomycin impregnated polymethyl-methacrylate (PMMA) bone cement spacer after two-stage exchange arthroplasty due to periprosthetic joint infection of the knee

**DOI:** 10.1186/1471-2334-14-144

**Published:** 2014-03-18

**Authors:** Jan Schmolders, Gunnar TR Hischebeth, Max J Friedrich, Thomas M Randau, Matthias D Wimmer, Hendrik Kohlhof, Ernst Molitor, Sascha Gravius

**Affiliations:** 1 Department for Orthopaedic and Trauma Surgery, Rheinische Friedrich-Wilhelms-University Bonn, Sigmund-Freud-Strasse 25, 53105 Bonn, Germany; 2 Institute for Medical Microbiology, Immunology, and Parasitology, Rheinische-Friedrich-Wilhelms Universität Bonn, Bonn, Germany

## Abstract

**Background:**

Periprosthetic joint infections (PJI) are often treated by two stage exchange with the use of an antibiotic impregnated spacer. Most of the two-stage exchange algorithms recommend the implantation of an antibiotic-impregnated spacer during the first stage for a period of 2–24 weeks before reimplantation of the new prosthesis. For the spacer to have a therapeutic effect, the local antibiotic concentration must be greater than the minimal inhibition concentration (MIC) against the pathogens causing the PJI. It must remain so for the entire spacer period, otherwise recurrence of infection or resistances might occur. The question as to whether a sufficient concentration of antibiotics in vivo is reached for the entire spacer period has not been answered satisfactorily.

**Case presentation:**

We here present a case of a histologically confirmed chronic PJI 20 month after primary arthroplasty. The primary knee arthroplasty was performed due to osteoarthritis of the joint. Initial assessment did not detect a causative pathogen, and two stage exchange with a vancomycin-gentamycin impregnated spacer was performed. At the time of reimplantation, sonication of the explanted spacer revealed a multi-resistant strain of staphylococcus epidermidis on the device and in the joint. Adaption of the therapy and prolonged treatment successfully eradicated the infection.

**Conclusion:**

According to the authors’ knowledge, the case presented here confirms for the first time the surface contamination (proven through sonication) of a vancomycin-/gentamicin- impregnated Vancogenx®-spacer with a MRSE after ten weeks of implantation.

This case study demonstrates the difficulties still associated with the diagnostics of PJI and the published different two stage treatment regimes with the use of antibiotic impregnated spacers.

## Background

The two-stage exchange arthroplasty remains the preferred surgical treatment for chronic periprosthetic joint infection (PJI) [1,2]. Most of the two-stage exchange algorithms recommend the implantation of an antibiotic-impregnated spacer during the first stage for a period of 2–24 weeks before reimplantation of the new prosthesis [3-5]. This procedure is supposed to provide an initially high local release of antibiotics, then decreasing continuously over time [6].

There have been numerous in-vitro studies investigating the release of different antibiotics from antibiotic-impregnated polymethyl-methacrylate (PMMA) spacers [7-9]. The question as to whether a sufficient concentration of antibiotics in vivo is reached for the entire spacer period has not been answered satisfactorily. Previous in vivo studies have shown that only a minor portion of the antibiotics incorporated in the bone cement is really eluted. Furthermore, some of these studies even show conflicting results [10,11]. The elution of antibiotics from the bone cement does not necessarily reflect the tissue concentrations. Different antibiotics are released at different rates and in a different way from the bone cement according to their physicochemical characteristics, maybe even causing synergistic effects and an improved elution when combining two water-soluble antibiotics in bone-cement [6].

The combination of vancomycin and an aminoglycoside (gentamycin or tobramycin) has a broad spectrum of activity, covering also a variety of gram-positive bacteria, such as MRSA and many staphylococcus epidermidis strains [6]. On the other hand, not all gram- negative bacteria are treated effectively [12]. Other combinations of antibiotics are also possible, but it must be kept in mind that missing antibiotics into the bone cement for custom made spacer is usually considered off-label use of the drug. Antibiotic-impregnated spacers are also commercially available (e.g. Vancogenx®, Merete GmbH, Berlin, Germany). They have, in contrast to custom made spacers, the advantage of a known chemical and mechanical characteristics [13,14].

For the spacer to have a therapeutic effect, the local antibiotic concentration must be greater than the minimal inhibition concentration (MIC) against the pathogens causing the PJI. It must remain so for the entire spacer period, otherwise recurrence of infection or resistances might occur. Therefore, the local antibiotic concentration in the tissue surrounding the spacer should be greater than the MIC previously determined for the pathogen that is responsible for the PJI [11,15].

In this case report, we describe the first known case of a foreign body infection, caused by a multi-resistant staphylococcus epidermidis (MRSE) on a gentamicin-vancomycin-impregnated spacer. The spacer was in situ for 10 weeks due to a chronic PJI of the knee, and the pathogen was detected through sonication after explantation of the spacer.

## Case presentation

We present the case of a 52-year old male patient with a histologically confirmed chronic PJI of the knee but without microbiological detection of a pathogen, undergoing a two-stage exchange arthroplasty in our hospital.

The diagnosis of PJI used in the presented case was modified to the criteria described by Zimmerli and Trampuz et al. [4,16,17].

A primary cemented knee arthroplasty with a gentamycin loaded bone cement was performed due to osteoarthritis of the joint. The initial postoperative period was uneventful, but the patient presented himself after 20 month with pain in the operated joint. Besides persistent swelling and local hyperthermia, there were no systemic signs of an infection (normal leukocyte count and normal serum level of C-reactive protein (CRP)). Patient’s comorbidities are a diabetes type II, a chronic obstructive bronchitis, obesity, a arterial hypertonica and a nicotine abuse.

A synovial joint fluid aspiration was performed under aseptic surgical conditions. Even after long-term culture incubation of 14 days, no pathogen was detected. The total synovial leukocyte count had been 353 cells/μl with a neutrophil percentage of 34% (PMN%).

Revision surgery of the knee joint was performed, revealing signs of an infection, including presence of purulence and infection-associated osteolysis, particularly in the medial and lateral femoral condyles.

Based on these findings, the present total knee prosthesis was explanted, thorough surgical debridement was performed, and a Vancogenx®-spacer (Merete Medical GmbH, Berlin, Germany) with vancomycin- and gentamycin supplements (1.8 g each per spacer) was implanted. Intraoperatively, tissue samples were taken from representative infected areas (from tibia and femur components, tibial and femoral medullar cavities, and joint capsules). Each of these tissue samples were divided in half, one part for microbiological, long-term incubation (10-14d), the other for histological analysis (classification of periprosthetic membranes according to Morawietz et al. [18]). From these five tissue samples, none showed positive for a pathogen in microbiology. Histologically, the periprosthetic membrane was classified to be of the infectious type (Morawietz type II [18]).

In accordance to the standards of our institution, and derived from an analysis of causative pathogens in our patients, an empirical antibiotic therapy was started, with vancomycin and piperacillin/tazobactam intravenously, and rifampicin orally, for 14 days.

The postoperative hospital stay was without further complications; after two weeks, therapy was changed to moxifloxacin and rifampicin orally and continued for an additional four weeks. Antibiotics were stopped and after another two weeks, joint fluid was aspirated under sterile surgical conditions. The cell count yielded 1380 Leukocytes/μl with 42% polymorph nuclear neutrophils, and microbiological testing in long-term incubation again turned out negative. After another two weeks, in total ten weeks after explantation, the same surgeon performed the reimplantation of a cemented rotating hinge knee prosthesis using vancomycin/gentamycin loaded cement for the undersurface (RT Plus Solution modular; Smith & Nephew, Marl, Germany). Intraoperatively, no macroscopic signs of infection could be detected; in particular, in contrast to the explantation, the joint fluid was clear and the bone quality, underneath the femoral as well as tibial spacer components, was no longer gave the impression of being infected. Again several tissue samples were taken as mentioned afore for microbiological incubation and histological examination. Additionally, the explanted spacer was sent in for sonication (BactoSonic® 14.2, Bandelin electronic GmbH & Co. KG, Berlin, Germany), as described by Trampuz et al. [19]. Postoperatively, antibiotics were started prophylactical, again with vancomycin and piperacillin/tazobactam intravenously and rifampicin p.o.

In the sonication fluid, a multi-resistant staphylococcus epidermidis (MRSE) was detected, also being resistant to rifampicin, as proof of a bacterial growth on the Vancogenx®-spacer. Identification was performed by MALDI-TOF (Biomérieux, Nürtingen, Germany) and antibiotic susceptibility was performed by VITEK-2 (Biomérieux, Nürtingen, Germany). The MIC values were interpreted susceptible by European Committee on Antimicrobial Susceptibility Testing (EUCAST, Version 2.0 2012) as follows: Doxycyclin (1 mg/l), vancomycin (1 mg/l), linezolid (2 mg/l) and daptomycin (≤ 0.5 mg/l).

This MRSE would later show in three of the five tissue samples from the reimplantation surgery as well. Because of the level of resistance, the antibiotic therapy was adapted and changed to daptomycin in the dosage of 10 mg/kg KG, administered for a total of 14 days intravenously. Afterwards, an oral therapy with Linezolid was continued for 28 more days. During the follow-up period of one year after reimplantation, the patient did not present any local or systemic signs of infection. Serum CRP and leukocyte count remain negative, making the patient currently “highly probably infection free”, according to the criteria of Laffer et al. [16].

### Discussion

According to the authors’ knowledge, the case presented here confirms for the first time the surface contamination (proven through sonication) of a vancomycin-/gentamicin- impregnated Vancogenx®-spacer with a MRSE after ten weeks of implantation.

In a patient presenting 20 month after the primary arthroplasty with a painful joint, PJI must be considered as a differential diagnosis, even when systemic signs of infection are missing. Infections at this time point can be either hematogenous acute infections or a chronic primary low-grade infection. The negative cell count in the synovial fluid, as well as the lack of a systemic inflammatory response is pointing towards a low grade infection. During the first revision, considering the local, intraoperative findings, the surgeon decided on explantation and two-step exchange.

The choice to use a Vancogenx®-spacer seems, not assuming the presence of a difficult-to-treat pathogen at that time, sensible. By combining vancomycin and gentamicin, the most common pathogens of a chronic PJI (among others, coagulase-negative staphylococcus and propioni bacteria) are covered and treated. Even though our own analysis shows a considerably high rate of multi-resistant coagulase-negative staphylococcus and app. 30% gram-negative pathogens in our patient collective, vancomycin and gentamycin remain the first line local treatment in our institution [20,21]. To avoid the rapid development of bacterial resistances, especially in staphylococci, antibiotic combination therapies should be favored over monotherapies.

For combining vancomycin and gentamicin, synergistic effects have already been proven with regard to efficacy and improved release kinetics in local drug delivery. The emergence of the highly resistant MRSE in the current case could be explained by an already existing gentamicin-resistant pathogen at the point of explantation. Especially the use of gentamicin-containing PMMA in the primary arthroplasty could induce such resistances through the sub-therapeutic release of the antibiotic over the 20 month period, as previously observed by Neut et al. [22]. If this was also the case in the patient presented herein cannot be determined from the records.

The negative microbiological findings in the preoperative joint aspiration, as well as in the intraoperative specimens from the explanation give rise to discussion, for the local site findings, the positive histology and the clear signs of an infection intraoperatively all clearly pointed to the presence of a PJI. PJI without bacterial growth are indicated in the literature with 10–26.5% [4,21]. The reasons for this are numerous. The patient did not receive any antibiotic therapy prior to the revision, ruling out this common source of false negatives. Other reasons include a low inoculum, adherent bacteria on the implant, difficult to identify bacteria (e.g. “small colony variants” (SCV)), as well as logistic (storage and transportation of the specimen) as well as technical problems, or insufficient testing in the laboratory [23]. The sonication of the primary implant–this procedure was, at that point, not yet available to our institution–might have improved the microbiological diagnostics. Trampuz et al. [19] proved that, compared to the culture of periprosthetic tissue samples alone, a significantly higher sensitivity can be reached through this method.

The second possibility for the emergence of the gentamicin-resistance of the pathogen is the rather long implantation time of ten weeks, inducing resistances through sub-therapeutic concentrations of the antibiotics in the surrounding tissue. Many acknowledged therapy regimes include an implantation time of antibiotic-impregnated spacer also in this length; nevertheless, the scientific data is mixed regarding the release of therapeutic substances over such a long time period. Most data on this matter is derived from in-vitro release kinetics. They do not, however, take into account the multifaceted interactions between the local anti-microbial therapy, the causative bacteria, the properties of the PMMA, the patient’s immune system and an additional systematic antibiotic therapy. More studies have proven high concentrations of antibiotics in the drainage fluid during the first few days after the implantation of an antibiotic-impregnated spacer [14,24,25]. Decreasing concentrations at more therapeutic doses could be documented up to 7 days after spacer implantation [14]. Masri et al. [10] showed a release of tobramycin and vancomycin from the spacer over a time period of an average of 118 days, Hsieh et al. [26] for vancomycin and azetreonam of an average of 107 days. Bertazzoni Minelli et al. [6] showed a high initial gentamycin- and also vancomycin-release in the early phase after implantation, followed by a lower, but constant release over the time period of 3–6 months. It must be critically noted that the previously named studies determine either the intraarticular antibiotic concentration or the in-vitro release of the spacer after explantation. In contrast, Fink and colleagues [11] were able to prove that therapeutic concentrations above the MIC inside of the periprosthetic tissue could be detected after an implantation time of 6 weeks both for copal cement (gentamycin and clindamycin supplement) as well as for copal cement spacer with vancomycin supplement. Again, in-vitro studies of explanted spacers also revealed major differences in the residual amount of antibiotics, in the effectiveness and in release characteristics at identical implantation times. The discrepancy of these results is still to a great extent unclear. The local tissue blood supply as well as the pH-value of the tissue are considered relevant factors, and also the covering of the surface of the spacer with scar tissue [14,27].

Therapy algorithms are clinically established and acknowledged; they include a sterile joint aspiration during the implant-free interval and after an antibiotic-free interval of at least two weeks. Only after negative results of long-term incubation, the reimplantation of the new prosthesis can be considered. This procedure has the decisive disadvantage that no systematic antibiotic protection is present at a time of continually decreasing local antibiotic release from the spacer. In the current case, over a time period of 4 weeks, oral, systemic antibiotics were stopped before the new prosthesis was reimplanted, while the spacer was already in place for six weeks. The value of this additional joint puncture must be critically discussed, since every fifth PJI is missed through joint aspiration alone, anyhow [28]. Anagnostakos et al. [14] even recommend an adaptation in dosage of the systematic antibiotic therapy, in order to avoid the creation of multi-resistant strains. Alternative therapy concepts include shorter time periods under continual i. v.-antibiotic therapy or the exchange of the antibiotic-impregnated spacer to ensure high local drug levels [4]. With the knowledge of the pathogen findings in the current case, a two-step process without spacer and the continuous intravenous application of antibiotics over a time period of 6 weeks, corresponding to published recommendations with multi-resistant bacteria, seems another sensible treatment option [4].

In the current case, rifampicin was used in the empirical therapy regime right after the explantation of the primary prosthesis. It is still unclear if the use of a biofilm-effective antibiotic (rifampicin, in the present case) makes sense combined with a spacer at all. With the removal of the prosthesis and extensive debridement, biofilms should be reduced to a minimum, and antibiotics such as vancomycin or piperacillin should also be effective. Clauss et al. [29] identified that the systemic rifampicin therapy, in combination with a spacer, is a possible cause for subsequent rifampicin-resistances (OR 7.9; p = 0.057). In the case presented here, this was also seen. It should therefore be considered to withhold the biofilm-effective antibiotics until reimplantation.

## Conclusion

In summary, the current case impressively presents the problems regarding the diagnosis and therapy of PJI. Differential diagnostics of a low grade infection can be difficult and different tests can yield conflicting results, including false negatives and false positives. New methods–like the sonication–can essentially improve perioperative diagnosis. The use of spacers includes advantages as well as disadvantages, and little is known about the in-vivo effect of the incorporated antibiotics. Different regimes in treatment and the perfect time point and setting for the reimplantation yet remain to be determined and tested in clinical practice. Prospective, controlled therapy studies are necessary to help solve these important clinical problems.

## Consent

Written informed consent was obtained from the patient for publication of this Case report and any accompanying images. A copy of the written consent is available for review by the Editor of this journal.

### Ethics statement

This study is in accordance with the Helsinki Declaration. No human experimentation was done. IRB approval for this case report was not required as per German regulations.

## Competing interest

SG has lecture activities for Merete Medical GmbH. None of the authors have a competing interest to disclose in relation with this work.

## Authors’ contributions

SJ, HGTR, FM, RTM, WMD, KH, ME and GS treated the patient and collected the primary data. SJ, FM, RTM and GS were responsible for drafting the manuscript. All authors read and approved the final version of the manuscript before submission.

## Pre-publication history

The pre-publication history for this paper can be accessed here:

http://www.biomedcentral.com/1471-2334/14/144/prepub
